# Biopreservative efficacy of *Enterococcus faecium*-immobilised film and its enterocin against *Salmonella enterica*

**DOI:** 10.1186/s13568-023-01516-z

**Published:** 2023-01-23

**Authors:** Muzamil Rashid, Sunil Sharma, Arvinder Kaur, Amarjeet Kaur, Sukhraj Kaur

**Affiliations:** 1 Department of Microbiology, Guru Nanak Dev University, Amritsar, Punjab India; 2 Department of Zoology, Guru Nanak Dev University, Amritsar, Punjab India

**Keywords:** Bacteriocin, Lactic acid bacteria, Salmonellosis, Sodium alginate films, Bio preservative

## Abstract

The growing awareness about the adverse health effects of artificial synthetic preservatives has led to a rapid increase in the demand for safe food preservation techniques and bio preservatives. Thus, in this study, the biopreservatives efficacy of enterocin-producing *Enterococcus faecium* Smr18 and its enterocin, ESmr18 was evaluated against *Salmonella enterica* contamination in chicken samples. *E. faecium* Smr18 is susceptible to the antibiotics penicillin-G, ampicillin, vancomycin, and erythromycin, thereby indicating that it is a nonpathogenic strain. Further, the enterocin ESmr18 was purified and characterised as a 3.8 kDa peptide. It possessed broad spectrum antibacterial activity against both Gram-positive and Gram-negative pathogens including *S. enterica* serotypes Typhi and Typhimurium. Purified ESmr18 disrupted the cell membrane permeability of the target cell thereby causing rapid efflux of potassium ions from *L. monocytogenes* and *S. enterica.* Chicken samples inoculated with *S. enterica* and packaged in alginate films containing immobilised viable *E. faecium* resulted in 3 log_10_ colony forming units (CFU) reduction in the counts of *S. enterica* after 34 days of storage at 7–8 °C. The crude preparation of ESmr18 also significantly (p < 0.05) reduced the CFU counts of *salmonella-*inoculated chicken meat model. Purified ESmr18 at the concentration upto 4.98 µg/ml had no cytolytic effect against human red blood cells. Crude preparation of ESmr18 when orally administered in fish did not cause any significant (p < 0.05) change in the biochemical parameters of sera samples. Nonsignificant changes in the parameters of comet and micronucleus assays were observed between the treated and untreated groups of fishes that further indicated the safety profile of the enterocin ESmr18.

**Supplementary Information:**

The online version contains supplementary material available at 10.1186/s13568-023-01516-z.

## Introduction

Poultry meat is the second highest in terms of global meat consumption (Chai et al. 2017) that makes it the major source of meat-associated food-borne illness. The most common infectious disease transmitted by meat is salmonellosis caused by *Salmonella enterica*. Salmonellosis includes gastroenteritis caused by non-typhoidal strains of *Salmonella* and chronic enteric fever i.e. typhoid, caused by *S. enterica* typhi and paratyphi (Ryan et al. 2017). Every year, 11–20 million people become sick due to typhoid out of which 128,000 to 161,000 people die (WHO 2018). Almost 6% of the treated typhoid patients become chronic carriers of *S. enterica typhi*, that shed the bacteria in faeces resulting in continued transmission of the disease (Trujillo et al. 1991). Further, Increasing incidences of antibiotic resistance has been observed among food-transmitted *Salmonella* spp. (Karkey et al. 2018). Thus, *Salmonella* contamination is one of the major challenges for poultry meat producers. The most common antimicrobial intervention used in chicken is refrigeration. But *Salmonella* spp. not only survives refrigeration temperature (Dominguez and Schaffner 2009) but also multiplies in chicken at temperature as low as 5 °C (Smadi et al. 2012). Treatment of chicken meat with chemical preservatives such as sodium nitrite and sodium benzoate (Sindelar and Milkowski 2011) is done to prevent lipid oxidation and inhibit the growth of microorganisms but nitrites could be carcinogenic due to their ability to form nitrosamines (Massey and Lees 1992) Sodium benzoate at different doses has number of adverse health effects (Piper and Piper 2017). Owing to these limitations, more attention has been drawn towards exploration of safe alternative bio preservative technologies.

Lactic acid bacteria (LAB) is known to prolong the shelf life of fermented food due to their ability to secrete number of metabolites such as organic acids, antimicrobial peptides known as bacteriocins (Mokoena et al. 2021) etc. Some genera of LAB such as *Lactobacillus* spp., *Pediococcus* spp, *Enterococcus* spp. etc. have been successfully used for the preservation of processed fruits and vegetables (Agriopoulou et al. 2020), cheese (Medved 'ová et al. 2020) dairy and meat (McMullen and Stiles 1996). As part of the starter culture, bacteriocin-producing *Enterococcus* spp. was shown to inhibit food pathogens in cheese (Giraffa 2003) and meat (Callewaert et al. 2000). The application of LAB for the preservation of non-fermented food products is a challenge because the addition of the bacterial strains to the food can change its sensory properties due to fermentation. Thus, to overcome this limitation, immobilisation of LAB in different matrices can be done. Immobilisation of *L. plantarum* in alginate films have been successfully used for the preservation of cheese (Silva et al. 2022). However, the preservative effect of immobilised LAB in meat has not been studied.

Bacteriocins have long been the focus of research as a potential replacement for chemical preservatives. They are being explored for many applications such as therapeutic drugs (Soltani et al. 2021) and as bio preservatives in food (Cleveland et al. 2001) and cosmetics (Maurício et al. 2017). Two bacteriocins i.e. pediocin produced by *Pediococcus acidilactici* (Papagianni and Anastasiadou 2009) and nisin produced by *Lactococcus lactis* (Deegan et al. 2006) have been approved by Food and Drug Administration for use as a food preservative. However, due to their inability to inhibit Gram-negative bacteria (Zhou et al. 2016) they have limited applications as food preservatives in chicken meat products. Enterocins are the bacteriocins secreted by *Enterococcus* spp**.** Some enterocins exhibit broad-spectrum activities against both Gram-negative and Gram-positive pathogens including *S. enterica* (Ankaiah et al. 2018). Several studies have shown the potential of enterocins in inhibiting *L. monocytogenes* in the cooked and raw meat (Kasimin et al. 2022). However, the biopreservative efficacies of bacteriocinogenic strain or its bacteriocin in inhibiting *S. enterica* in meat models have not been evaluated.

Therefore, in this study, we explored the potential of enterocin-producing *E. faecium* Smr18 and its enterocin ESmr18 in inhibiting *S. enterica* in raw chicken. Further, we purified the enterocin ESmr18 from the culture supernatant of *E. faecium* and tested its stability at refrigeration temperature. The biopreservative efficacy of ESmr18 against *S. enterica*-inoculated chicken was also tested. The safety of ESmr18 was evaluated in in vitro and in vivo studies.

## Material and methods

### Bacterial isolates

*Enterococcus faecium* Smr18 was received from Dr. Sukhraj Kaur's laboratory. It was isolated from the swab samples of healthy vaginal microflora of woman after obtaining her written informed consent. The study was approved by the Human Ethics Committee of Guru Nanak Dev University, Amritsar, India. *E. faecium* was cultured in de Man Rogosa and Sharpe (MRS, Himedia Laboratories Pvt. Ltd., Mumbai, India) broth at 37 °C in anaerobic jars under stationary conditions. For conducting the experiments*, E. faecium* was propagated twice in MRS medium at 37 °C. All the chemicals used in the study were purchased from Himedia, except where specifically mentioned.

The strain was identified by using partial sequencing of 16sRNA done at National Centre for Cell Science, Pune, India. The sequence so obtained was compared with the known sequences of other *Enterococcal* spp. aligned by using National Center for Biotechnology Information—Basic Local Alignment Search Tool (NCBI-BLAST) database. Phylogenetic tree was constructed by using MEGA 6 software following Neighbourhood Joining method and Kimura2 Gamma I model. The strain was deposited to Microbial Type Culture Collection (MTCC), Institute of Microbial technology, Chandigarh, India with MTCC number 13248.

The pathogenic bacterial strains used in the study and procured from National Collection of Industrial Microorganisms (NCIM), Pune, India were *Listeria monocytogenes* NCIM 5277, *Staphylococcus aureus* NCIM 5718, *Pseudomonas aeruginosa* NCIM 2862, *Shigella flexneri* NCIM 5265, *Klebsiella pneumoniae* NCIM 5215 and *Escherichia coli* NCIM 5662. *S. enterica* serotype Typhi MTCC 733, S. *enterica* serotype Typhimurium MTCC 1251, *S. enterica* serotype Typhimurium MTCC 1252 and *Streptococcus pyogenes* MTCC 1927 were procured from MTCC. The pathogenic indicator bacteria were propagated at 37 °C under aerobic conditions in Brain heart infusion (BHI) broth.

### Preparation of enterocin and its susceptibility to various enzymes

Purification of the enterocin ESmr18 was done by ammonium sulphate precipitation of cell-free culture supernatant (CS) of *E. faecium* followed by cation exchange chromatography. The proteins were precipitated from the CS by adding ammonium sulphate at 60% saturation (w/v) and mixing it on magnetic stirrer at 4 °C, for overnight. The precipitated proteins were separated by centrifugation (8000*g*; 10 min) and dissolved in sodium acetate buffer (20 mM; pH 4.5). The desalting of the precipitates was done by using Biogel PD-10 column (GE Health Care, USA) and the active fractions from the PD-10 column were pooled and referred as crude ESmr18.

For preparation of the purified ESmr18, the pooled fractions from PD-10 column were loaded onto SP-Sepharose Fast Flow cation-exchange column (5010 mm; GE Health Care) and the bound proteins were eluted by using linear gradient of 0.1 to 1 M NaCl. The active fractions were lyophilized and dissolved in distilled water. The purity of the protein was evaluated by electrophoresis on a 17% denaturing polyacrylamide gel and the protein concentration was evaluated by Bradford’s method (Bradford 1976).

Further, the susceptibility of various enzymes on the antimicrobial activities of crude and purified ESmr18 was determined. CS and ESmr18 were treated with enzymes proteinase K, trypsin, pepsin, and lipase (Sigma Aldrich, India) at the concentration of 1 mg/ml for 1 h at 37 °C, followed by heat inactivation at 60 °C for 10 min. The residual antimicrobial activity was determined by agar gel diffusion assay.

### Antimicrobial activity

The antimicrobial activity of the CS of *E. faecium*, and the purified enterocin ESmr18 was determined against various pathogenic bacterial strains by using agar gel diffusion assay (Geis et al. 1983). CS was prepared by centrifuging the overnight culture of *E. faecium* Smr18 at 8000*g* for 10 min at 4 °C and then passed through syringe filters (0.22 µm) and kept at 4 °C till further use. For conducting agar gel diffusion assay the optical density (OD; at 550 nm) of pathogenic bacteria in log phase was adjusted to 0.1 and 100 µl of the culture was distributed onto BHI agar medium plates. A cork borer was used to cut wells in the agar plates with a diameter of 6.0 mm. Thereafter, 100 µl of CS (pH 6.5) crude extract and purified ESmr18 were added to the wells, and the plates were incubated at 4 °C for 4 h to allow the samples to diffuse. The plates were then incubated at 37 °C under aerobic conditions. After 24 h, the zones of inhibition were measured in millimetres.

### Immobilization of *E. faecium* in films and antimicrobial activity of the films

Viable *E. faecium* Smr18 cells were immobilised in a sodium alginate film. The film was prepared by mixing sodium alginate (4% w/v), agar (3% w/v) and glycerol (20% v/v) in distilled water for 15 min on a magnetic stirrer at ambient temperature. The mixture was then sterilised by boiling for 20 min in a water bath. The solution so formed was mixed with autoclaved MRS medium in the ratio 1:1 under sterile conditions. 10 ml of the mixture was poured in Petri plates and allowed to cool down to semi-solid state before adding viable *E. faecium* cells (5 × 10^8^ CFU). The plates were left undisturbed for 20 min. After 20 min, 20 ml of 2% calcium chloride solution was added for the polymerisation of sodium alginate film and the plates were again left undisturbed for 15 min. The extra calcium chloride was discarded, and the films were allowed to dry. Another film prepared by following similar process but without *E. faecium* cells was used as negative control. For determining the antimicrobial activity, films were cut with the help of well borer and placed on BHI agar plates inoculated with 100 µl of the overnight grown culture of *S. enterica* (OD set at 0.1)*.* The plate was kept at 37 °C for 24 h and clear zones were measured.

The bio preservative efficacy of the film was tested against *S. enterica*-inoculated chicken model. Fresh boneless chicken (500 g) was procured from the local market and autoclaved for 10 min for complete sterilization. Overnight cultured *S. enterica* cell suspension containing 6 × 10^7^ CFUs/g was added to the pieces*.* The chicken pieces were covered with *E. faecium*-immobilised film and film without *E. faecium* in separate Petri dishes and stored at 7–8 °C. The pieces (1 g) were removed at different time intervals and plated on *Salmonella Shigella* agar (SS agar) containing plates for CFU counting.

### Efflux of potassium ions

To determine the mechanism of action of enterocin, we evaluated the effect of ESmr18 on the stability of the cell membrane of *S. enterica* MTCC 733 and *L. monocytogenes* NCIM 5277. Disruption of the cell membrane by the action of enterocin may result in efflux of small ions from the cell. Therefore, we evaluated the effect of ESmr18 treatment of pathogens at minimum inhibitory concentration (MIC) values on the extracellular potassium ion concentration (McAuliffe et al. 1998). The bacterial cells in mid-log phase were harvested by centrifugation at 8000*g* for 5 min to obtain cell pellet. The pellet was washed twice and re-suspended in 2.5 mM sodium HEPES (4-(2-hydroxyethyl)-1-piperazineethanesulfonic acid) buffer (pH 7.0) at OD_600_ 1.0. Purified ESmr18 was added to the cell pellets of *S. enterica* and *L. monocytogenes* in two separate tubes to obtain final concentrations 3.2 µg/ml. Samples (1 ml) were taken at different time intervals and immediately chilled on ice. *S. enterica* and *L. monocytogenes* cells in HEPES buffer without ESmr18 was used as controls. The samples were filter sterilised (0.2 µ) to separate the cells and the potassium ion concentration in the supernatants was determined by flame photometry (Systronics 128, Gujarat, India). The experiment was performed thrice in triplicates.

### Shelf life, stability, and bio preservative effect of crude ESmr18

Before the bio preservative effects of the crude preparation of ESmr18 was tested in chicken samples, it is important to study the shelf life of ESmr18 dissolved in water and sodium acetate buffer. The crude ESmr18 dissolved in distilled water and sodium acetate buffer (pH 4.5) were stored at refrigeration conditions (7–8 °C) for 6 months. At different time points twofold dilutions of the two samples were tested for its antimicrobial activity against *S. enterica* by agar gel diffusion assay in terms of arbitrary units (AU)/ml. AU is defined as the reciprocal of the highest dilution that showed zone of inhibition.

The stability of CS and crude Esmr18 at different pH and temperature treatments were evaluated. CS and crude ESmr18 were exposed to different temperatures (60, 80 and 100 °C) for upto 90 min and autoclaving for 40 min. The residual antimicrobial activity was determined by using agar gel diffusion assay. To determine the effect of pH, the pH of CS and crude ESmr18 was adjusted to different values ranging from 2 to 10 and incubated at 37 °C for 1 h. Thereafter, the pH was reset to 6.5 and the residual antimicrobial activity was determined by using agar gel diffusion assay.

The bio preservative effect of crude ESmr18 was determined on the chicken meat inoculated with *S. enterica*. Fresh boneless chicken meat was purchased from a local vendor in Amritsar, India. Fresh boneless chicken (500 g) was procured from the local market and autoclaved for 10 min for complete sterilization. Overnight cultured *S. enterica* cell suspension containing 6 × 10^7^ CFUs/g was added to the pieces and then crude ESmr18 (15 µg/g). For vehicle control, sodium acetate buffer was used. The counts of *Salmonella* in different samples were quantified at different time points over a period of 35 days by plating on SS agar plates.

### Hemolysis assay

Some bacteriocins are known to have toxicity against host cells. Therefore, we tested the hemolytic activity of ESmr18 against human red blood cells (RBCs) by using haemoglobin release assay (Paiva et al. 2012). For the preparation of RBCs, blood was drawn from persons over the age of 18 after obtaining their written informed consent. The protocol was approved by the Institutional Human Ethics Committee, Guru Nanak Dev University, Amritsar, and the study was carried out as per the guidelines of the Ethical Committee. Defibrinated human blood was centrifuged at 135*g* for 15 min at 37 °C and the RBC-containing pellet was suspended in 10 ml of phosphate-buffered saline (PBS; pH 7.2). RBC suspensions (500 µl) were treated for 1 h at 37 °C with 100 µl of various concentrations of ESmr18. The suspensions were then centrifuged for 5 min at 825*g*, and the haemoglobin release in the supernatant was measured at OD 415 nm. TritonX-100 (1%)-treated RBCs and PBS-treated RBCs were used as positive and negative controls, respectively. The percentage RBC lysis was calculated by using equation:

$$\left( {{\text{OD}}_{{\text{T}}} {-}{\text{OD}}_{{\text{C}}} } \right)/\left( {{\text{OD}}_{{\text{X}}} {-}{\text{OD}}_{{\text{C}}} } \right) \, \times {1}00.$$

OD_T_ is OD_415_ of ESmr18-treated RBCs; OD_C_ is OD_415_ of PBS-treated RBCs and OD_X_ is OD_415_ of 1% triton-treated RBCs.

### Safety evaluation of ESmr18 in fish

The use of ESmr18, warrants oral consumption, therefore it is important to determine the in vivo effects of the orally administered ESmr18. The in vivo effects of crude ESmr18 were evaluated in healthy *Cirrhinus mrigala.* The fishes having average length of 15–18 cm and average weight of 90–100 g were acquired from the Government Fish Farm, Rajasansi, Amritsar. They were transported to the lab and placed directly in acclimation tanks with tap water temperature at 24.8 ± 0.32 °C, dissolved oxygen 6.4 ± 0.09 mg/L, total dissolved solids 133.3 ± 2.33 mg/L, electrical conductivity 457 ± 1.15 S/cm and pH 7.01. During the acclimatisation and testing phases, the photoperiod was kept at a regular 12 h light–dark cycle. Throughout the trial, fish were given commercial fish food (fishmeal, vegetable proteins, and binding agents such as wheat) ad libitum at a rate of 2% of body weight. The test water was changed daily 1 h after feeding the fish.

To study the biosafety of crude ESmr18, 700 µg of crude ESmr18 was orally administered to a group of 6 fishes. The vehicle treated (VC) group was administered 200 µl of sodium acetate buffer (pH 4.5). The third group was left untreated (UT). The fishes were monitored for any behavioural change before and during the experiment. After 96 h fishes were sacrificed, the liver, kidney, and blood were taken and used in the comet assay. Blood was taken through cardiac puncture.

### Comet assay

DNA damage in the blood, liver, and kidney of the fishes in the treated, VC and UC groups was determined by using comet test (Yun et al. 2014) with minor changes. Slides covered with 1% normal melting point agarose were layered with 0.75% low melting point agarose containing blood, liver, and kidney cells and allowed to settle at 4 °C. The slides were subsequently submerged for 2 h at 4 °C in cold lysing buffer (2.5 M NaCl, 100 mM EDTA, 0.25 M tris aminomethane, 0.25 M NaOH, 1% triton X-100, 10% DMSO, pH 10.0). The slides were then coated again with 0.5% normal melting point agarose and allowed to solidify. Electrophoresis was carried out for 20 min at 25 V and 300 mA after the slides were coated with electrophoresis buffer (1 mM EDTA and 300 mM NaOH; pH 13). The slides were neutralised with 0.4 M Tris amino methane (pH 7.5) for 15 min, dried and stained with 20 μg/ml ethidium bromide. Analysis of the slides were done by fluorescence microscope (Nikon ECLIPSE E200) and images shot with Nikon D5300 camera. For each treatment group, 100 cells per sample were scored in triplicate. Various parameters like tail length (TL), tail moment (TM), and % tail DNA were calculated using Casplab Software.

### Micronucleus test

Homogenous blood smear of fish was prepared on a clean glass slide and air-dried for half an hour at room temperature. The slides were fixed in methanol, stained with 5% Giemsa dye for 15–20 min, and 1000cells/group were scanned at 100× by using light microscope (Olympus scanner; CX31) for evaluating any nuclear or cellular abnormalities.

### Statistical analysis

All the experiments were carried out in triplicates, and bars depict means ± SD standard deviation. To determine differences between mean values of different groups, a one-way analysis of variance (ANOVA) was used. The different treatment groups were compared by using the Tukey's test and the level of significance was set at 5% (p < 0.05). The software SPSS version 16.0 (SPSS Inc., Chicago, IL, USA) was used for statistical analysis.

## Results

### Bacterial identification

*E. faecium* Smr18 was selected for this study because of its broad-spectrum antimicrobial activities against both Gram-positive and Gram-negative pathogens. The isolate was identified by sequencing 16srRNA gene, and the sequence was submitted to NCBI database with accession no. OK598049. Phylogenetic tree was constructed based on 16SrRNA sequence that showed close similarity to *E. faecium* type strain LMG 11423 (Additional file 1: Fig. S1).

### Antimicrobial activity of crude and purified ESmr18

Purification of ESmr18 from cell free CS of *E. faecium* Smr18 was done using ammonium sulphate precipitation followed by cation-exchange chromatography. The active fractions were lyophilized and subjected to sodium-dodecyl polyacrylamide gel electrophoresis (SDS-PAGE) analysis to ensure purity of the protein. The concentrated purified fractions resolved as a single band with a molecular weight of around 3.8 kDa on SDS-PAGE (Additional file 1: Fig. S2).

The CS, crude and the purified ESmr18 of the *E. faecium* Smr18 had similar spectrum of antimicrobial activities as all of them were active against both Gram positive pathogenic bacteria (*L. monocytogenes, St. pyogenes*) and Gram negative pathogens, (*S. enterica* serotype Typhi and *S. enterica* serotype Typhimurium; Table 1). They exhibited no activity against rest of the tested pathogenic bacterial strains listed in the Table 1.

**Table 1:** Antimicrobial activity of the CS, crude and purified ESmr18 of *E. faecium* against various pathogenic indicator bacteria

S. no	Indicator bacteria	Strain	Zone of inhibition		
			CS	Crude ESmr18	Purified ESmr18
1	*L. monocytogenes*	NCIM5277	S^*^	S+	S
2	*Streptococcus pyogenes*	MTCC1927	S	S+	S
3	*S. enterica* serotype Typhi	MTCC733	S	S+	S
4	*S. enterica* serotype Typhimurium	MTCC1251	S	S+	S
5	*S. enterica* serotype Typhimurium	MTCC1252	S	S+	S
6	*Shigella flexneri*	NCIM5265	R	R	R
7	*Escherichia coli*	NCIM5662	R	R	R
8	*Klebsiella pneumonia*	NCIM5215	R	R	R
9	*Pseudomonas aeruginosa*	NCIM2862	R	R	R
10	*Staphylococcus aureus*	NCIM5718	R	R	R

^*^S Sensitive

S+ zone of inhibition > 15 mm

S zone of inhibition < 15 mm

R Resistant No zone of inhibition

The antibacterial activities of the cell free culture supernatant (CS), crude extract, and purified ESmr18 were evaluated by agar well diffusion assay. The pH of the CS was adjusted to 6.5 with 1 N sodium hydroxide before evaluation of antimicrobial activity. The experiment was carried out in triplicates.

### Effect at various enzymatic treatment

**Fig. 1:**
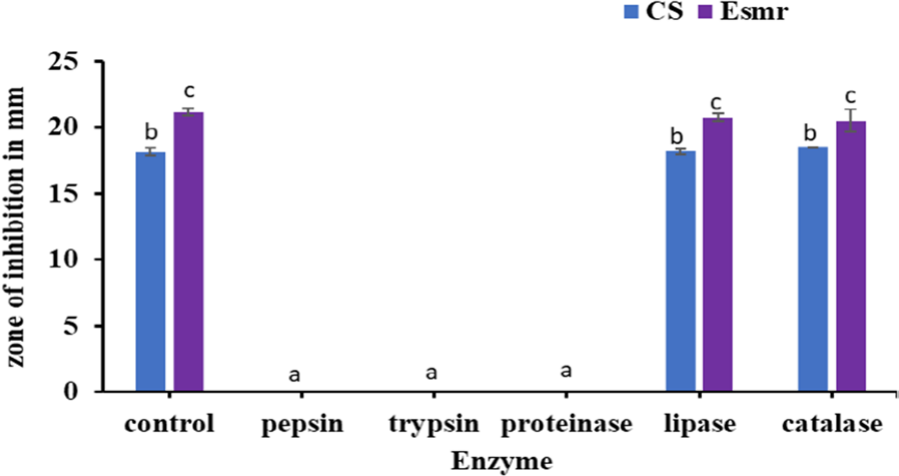
Effect of various enzymes on the antimicrobial activity of CS and purified ESmr18. CS and ESmr18 were treated with 1 mg/ml of various enzymes for 1 h and the residual antimicrobial activity was tested by agar well diffusion assay after heat-inactivation of the enzymes. The untreated CS and ESmr18 were used as control. Error bars are representative of mean ± SD of the three independent experiments performed in triplicates. Letters a, b and c denote significant differences at p < 0.05

Treatment of CS and ESmr18 with various enzymes was done to determine the biochemical nature of the antimicrobial activity. The antimicrobial activity of both CS and purified ESmr18 was completely inactivated after treatment with proteolytic enzymes pepsin, trypsin, and proteinase-k whereas, lipase and catalase had no effect on the antimicrobial activity (Fig. 1).

### Effect of Esmr18 on the cell membrane permeability

**Fig. 2:**
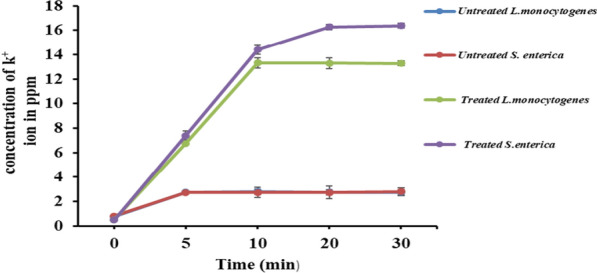
Efflux of potassium ions from *S. enterica* and *L. monocytogenes* cells after the treatment of different concentrations of ESmr18. Untreated *S. enterica* and *L. monocytogenes* cells were used as controls. Error bars are representative of ± SD of the three independent experiments performed in triplicates

The mechanism of bactericidal effect of bacteriocins is mostly explained by their ability to interact with the cell membrane and form pores. Thus, the effect of ESmr18 on the cell membrane permeability was evaluated by determining the efflux of potassium ions. As shown in Fig. 2, treatment of *S. enterica* and *L. monocytogenes* cells with ESmr18 resulted in significant (p < 0.05) increase in extracellular concentration of potassium ions at all time points as compared to the untreated control cells. The increased extracellular concentration of potassium ions was observed 5 min after the addition of ESmr18 to the cells of both the pathogens. The peak potassium ion concentrations of 14.5 and 16.3 ppm were observed in *L. monocytogenes* and *S. enterica* at 10 and 20 min, respectively, after which the effect plateaued (Fig. 2).

### Bio preservative efficacy of *E. faecium*-immobilised alginate film against *S. enterica*

For testing the use of *E. faecium* Smr18 cells as food preservative, the viable cells were immobilised in sodium alginate film. The film so produced was translucent, flexible, cohesive and had smooth appearance. A section of the film was tested for antimicrobial activity by using agar spot assay. The film containing *E. faecium* cells formed zone of inhibition against *S. enterica*, whereas film without *E. faecium* cells exhibited no antimicrobial activity against *S. enterica* cells (Additional file 1: Fig. S3).

**Fig. 3:**
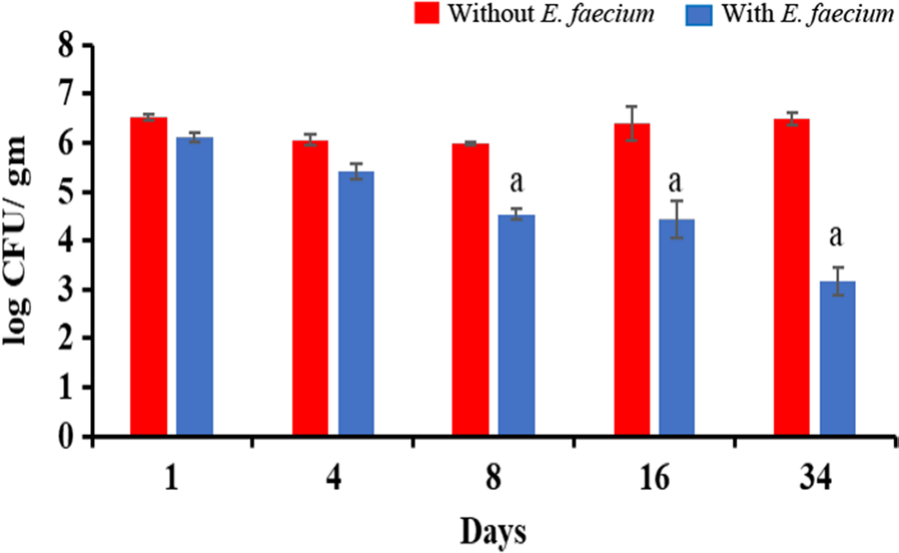
Viable counts of *S****.**** enterica* in chicken samples when stored at 7–8 ºC covered with films with and without viable cells of *E. faecium*. Error bars are representative of mean ± SD of the three independent experiments performed in triplicates. ^a^Indicates significant (p < 0.05) difference between *E. faecium* and without *E. faecium*-treated samples of the same day

Further, the antimicrobial activity of *E. faecium*-containing film was tested against *S. enterica*-inoculated chicken sample. Our results showed that on day 4, *E. faecium*-immobilised films reduced the CFU counts of *S. enterica* by 0.6 log_10_ as compared to films without *E. faecium*. On day 8 and day 16, the difference in the CFU_10_ counts in both the samples further increased by 1.3 and 1.75 log_10_. Maximum reduction in the *S. enterica* counts was obtained on day 34, when the number of CFUs decreased by 3.0 log_10_ CFU in the *E. faecium*-immobilised films as compared to film without *E. faecium* (Fig. 3).

### Stability of the antimicrobial potential of crude enterocin

**Fig.4:**
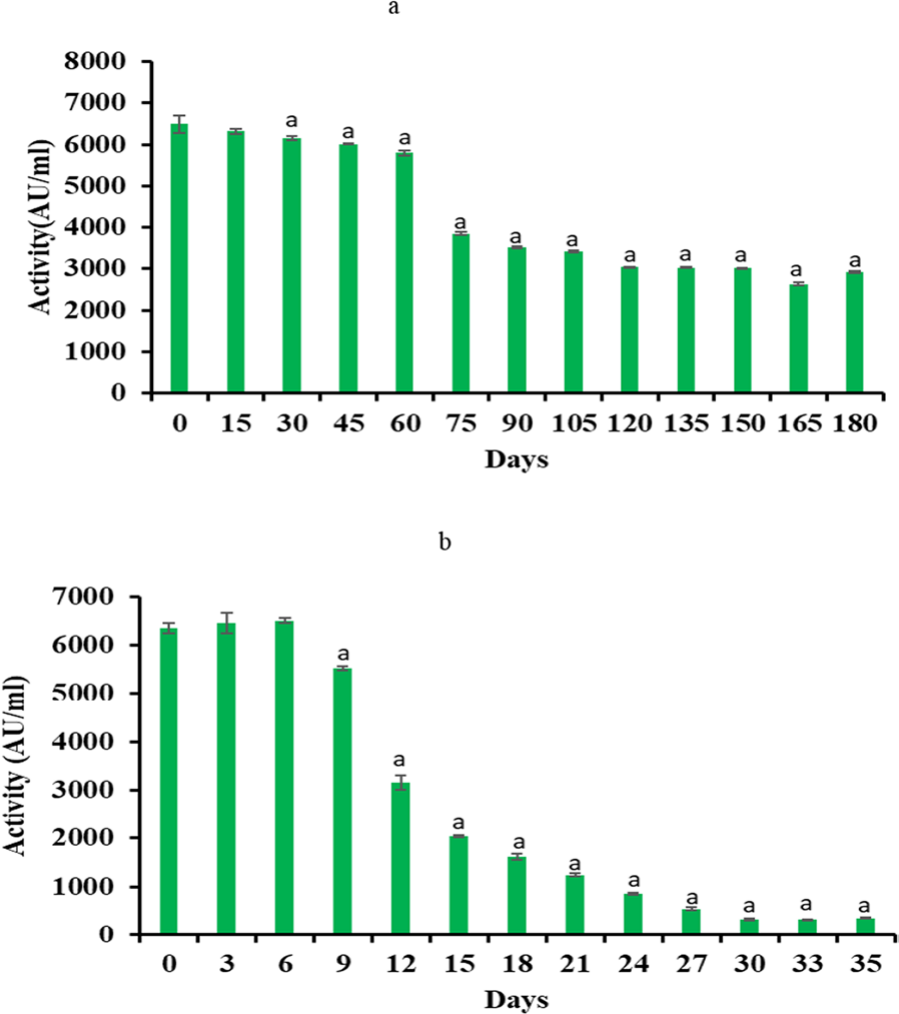
Stability profile of crude ESmr18 stored at (7–8 °C) (**a**) in distilled water (**b**) sodium acetate buffer and tested in terms of its antimicrobial activity against *S. enterica*. Error bars are representative of mean ± SD of the experiment performed in triplicates. ^a^Indicates data significantly (p < 0.05) different from the activity on day 0

Stability of the crude ESmr18 dissolved in distilled water and in sodium acetate buffer (pH 4.5) was determined at 7–8 ˚C at different time points. The antimicrobial activity of the crude ESmr18 dissolved in distilled water remained stable till 6th day (6488 AU/ml). On 12th day of storage, the antimicrobial activity was reduced by 50% and after 24 days the activity became negligibly low (Fig. 4a). On the other hand, in sodium acetate buffer, the activity remained stable till 60 days of storage at 7–8 °C. After 60 days, the antimicrobial activity decreased to half and remained constant till 180 days (Fig. 4b).

The stability of CS and crude ESmr18 at different pH and temperature was determined. Results in Additional file 1: Table S1 showed that both CS and Esmr18 retained the antimicrobial activities at pH 4, 6 and 8; however, at acidic pH 2 and at alkaline pH 10, both lost their activities. Further, temperature stability of CS and crude ESmr18 was tested by exposing to different temperature treatments for 90 min. Crude Esmr18 was stable at temperatures as high as autoclaving (Additional file 1: Table S1); however, at temperature above 80 °C the activity was reduced as shown by smaller zones of inhibition. On the other hand, CS was stable at temperature 60, 80 and 100 °C but not to autoclaving.

### Preservative effect of crude ESmr18 of on *S. enterica*-inoculated chicken meat

**Fig. 5:**
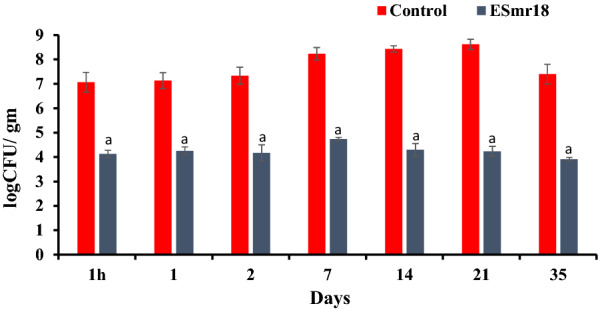
The viable cell counts of *Salmonella* in *S. enterica*-inoculated chicken meat samples treated with sodium acetate buffer (vehicle) and crude ESmr18 stored at 7 °C. Error bars are representative of mean ± SD of the three independent experiments performed in triplicates. ^a^Indicates significant (p < 0.05) difference on different days when compared with respective controls

The CFU counts of *S. enterica* were determined in fresh chicken meat samples inoculated with 7.0 log_10_ CFU/g of *S. enterica* cells in the presence or absence of crude ESmr18 at different time points. In untreated chicken meat samples, the counts of *S. enterica* increased by 1.6 log_10_ CFU/g on day 7 as compared to the initial count of 7.0 log_10_ CFU/g and they peaked (8.5 log_10_ CFU/g) on day 21, followed by 1 log_10_ decrease on day 35. However, in the case of ESmr18-treated samples, the *Salmonella* counts decreased by 3.0 log_10_ CFU as soon as 1 h after the addition of the enterocin. The differences in the counts of *S. enterica* in ESmr18-treated and untreated controls further increased to 3.75 log_10_ CFU on day7, 4.7 log_10_ CFU on day 21 and 3.9 log_10_ CFU on day 35 (Fig. 5).

### Evaluation of biosafety of crude ESmr18 in vitro and in vivo

The safety evaluation of ESmr18 was done in vitro by using hemolytic assay against human RBCs. When compared to the phosphate buffer saline (PBS)-treated negative control, purified ESmr18 at the maximum dose of 4.98 µg/ml caused no significant hemolysis of RBCs (Additional file 1: Fig. S4). On the other hand, treatment of RBCs with 1% Triton X-100 (positive control) resulted in 98% hemolysis.

The acute toxicity of crude ESmr18 in *C. mrigala* was determined after oral administration of the enterocin for four days. No mortality was observed in any of the groups till 96 h exposure. In addition, no stress indicators, such as anorexia, lethargy, exophthalmia, irregular swimming, gasping at the surface, skin irritations, or changes in body colour were observed in any of the groups. The fishes in all the groups swam actively throughout the experiment.

### Biochemical analysis

Liver and kidney function tests were conducted on the sera of orally administered ESmr18, VC and UC groups (Table 2). There was a non-significant (p < 0.05) difference in the liver and kidney parameters between the ESmr-treated, VC and UC groups. In ESmr18 group, the % change over control was maximum (− 16%) for protein and minimum (0%) for bilirubin. Whereas, in the VC group, % change over control was highest for Direct (D) bilirubin (30%) and minimum (0%) for creatinine.

**Table 2:** Biochemical analysis of serum samples of *C. mrigala*

Group	SGPT U/L	SGOT U/L	ALP U/L	T. Billuribin mg/dl	D. Bilirubin mg/dl	Protein g/dl	Urea mg/dl	Creatinine mg/dl	Albumin g/dl
UT	7.3 ± 0.95	76 ± 6.24	97.33 ± 3.50	3.1 ± 0.2	0.73 ± 0.15	2.66 ± 0.57	12.36 ± 1.75	0.26 ± 0.025	0.93 ± 0.152
VC	7.4 ± 0.99 (+ 1.36%)	76.65 ± 3.5 (+ 0.85%)	96.33 ± 3.20 (− 1.02%)	2.9 ± 0.4 (− 6.45%)	0.95 ± 0.22 (+ 30%)	2.33 ± 0.29 (− 12%)	12.66 ± 1.10 (+ 12.42%)	0.26 ± 0.011 (0%)	0.90 ± 0.1 (− 3.22%)
ESmr18	7.52 ± 1.0 (+ 3.01%)	73.63 ± 4.5 (− 3.11%)	97.66 ± 6.65 (+ 0.33%)	2.8 ± 0.55 (− 9.6%)	0.73 ± 0.15 (0%)	2.23 ± 0.30 (− 16.16%)	13.4 ± 0.65 (+ 8.41%)	0.24 ± 0.005 (− 7.69%)	1.03 ± 0.057 (+ 10.75)

Data expressed as mean ± S.D. (n = 6). UT: Untreated healthy control; VC: vehicle control; ESmr18: crude ESmr18 treated. Values in parenthesis are percent change over control

− indicates decrease over control

+ indicates increase over control

### Comet assay

**Fig. 6:**
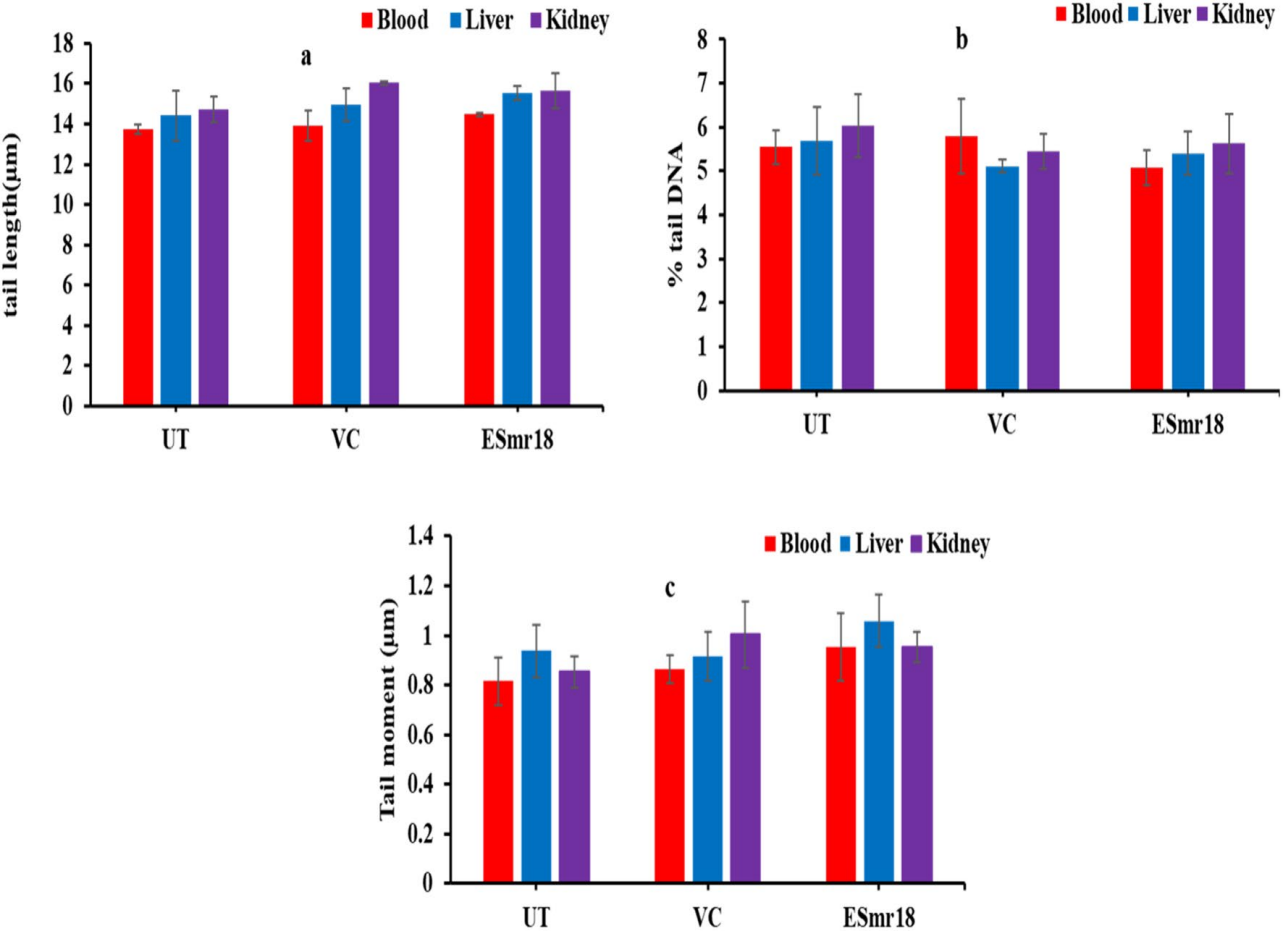
Effect of crude ESmr18 on tail length (**a**) % tail DNA (**b**) and tail moment (**c**) in blood, liver, and kidney cells of *C. mrigala*. Error bars are representative of mean ± SD of the experiment performed in triplicates. ^a^Indicates significant (p < 0.05) difference of the treated groups when compared with control

**Fig. 7:**
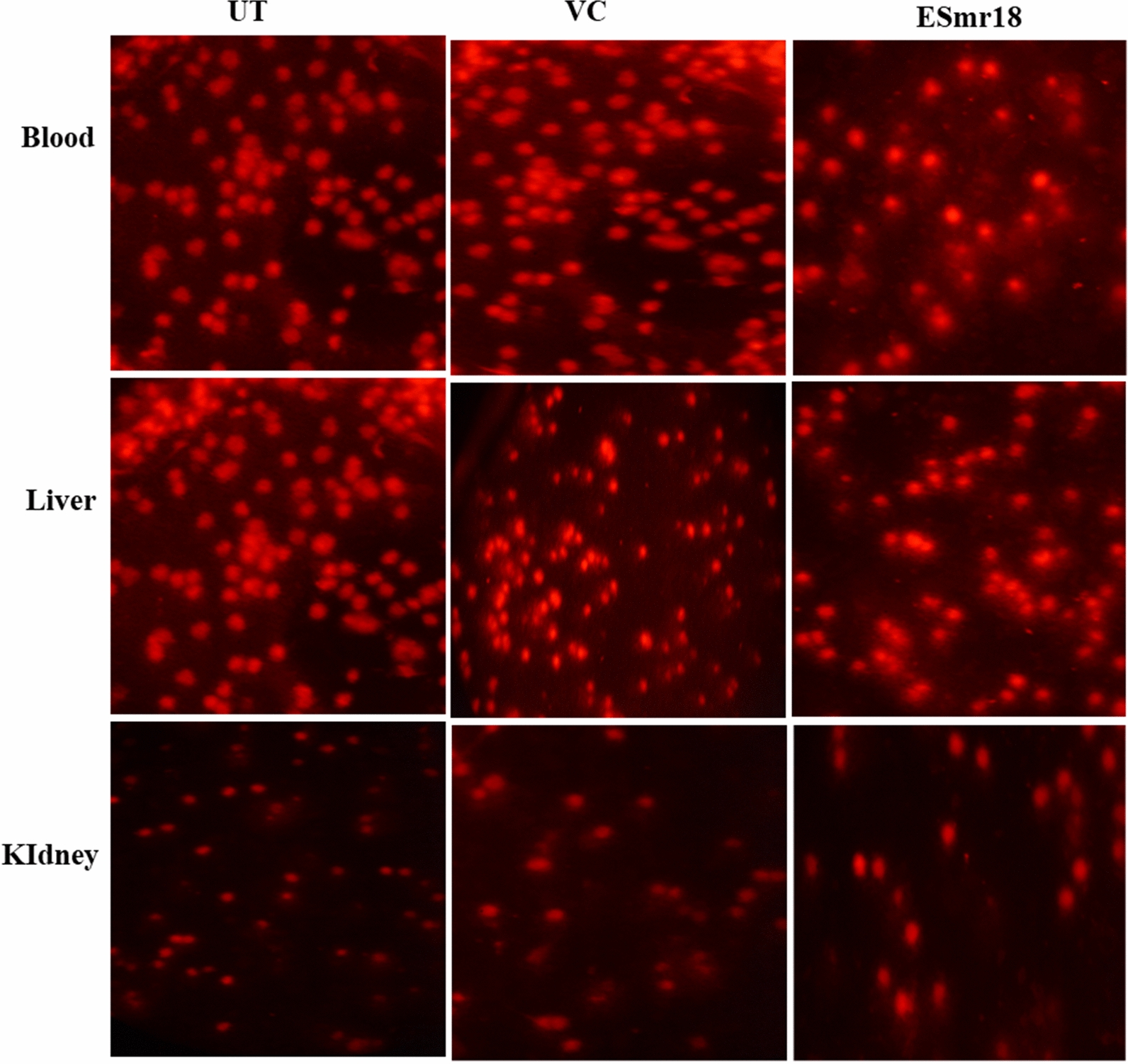
Comet assay of the blood, liver, and kidney cells of *C. mrigala.* UT: untreated; VC: vehicle control; ESmr18; crude ESmr18 treated. Error bars are representative of mean ± SD of the experiment performed in triplicates

Some strains of enterococci are known to secrete cytolytic proteins that may show DNA damage (York 2022). Therefore, the in vivo genotoxicity of the crude ESmr18 was determined by performing comet assay on blood, liver and kidney cells of *C. mrigala.* Three parameters i.e., TL, % tail DNA and TM were determined for assessing the DNA damage. There were non-significant differences in the average values of TL (Fig. 6a), tail DNA (Fig. 6b) and TM (Fig. 6c) between the 3 groups i.e., UT, VC and ESmr18-treated. Highest values of TL and TM were observed in kidney of VC group and liver of ESmr18 group, respectively. The microscopic images of the blood, liver and kidney cells do not show any cell damages (Fig. 7).

### Micronucleus test

**Fig. 8:**
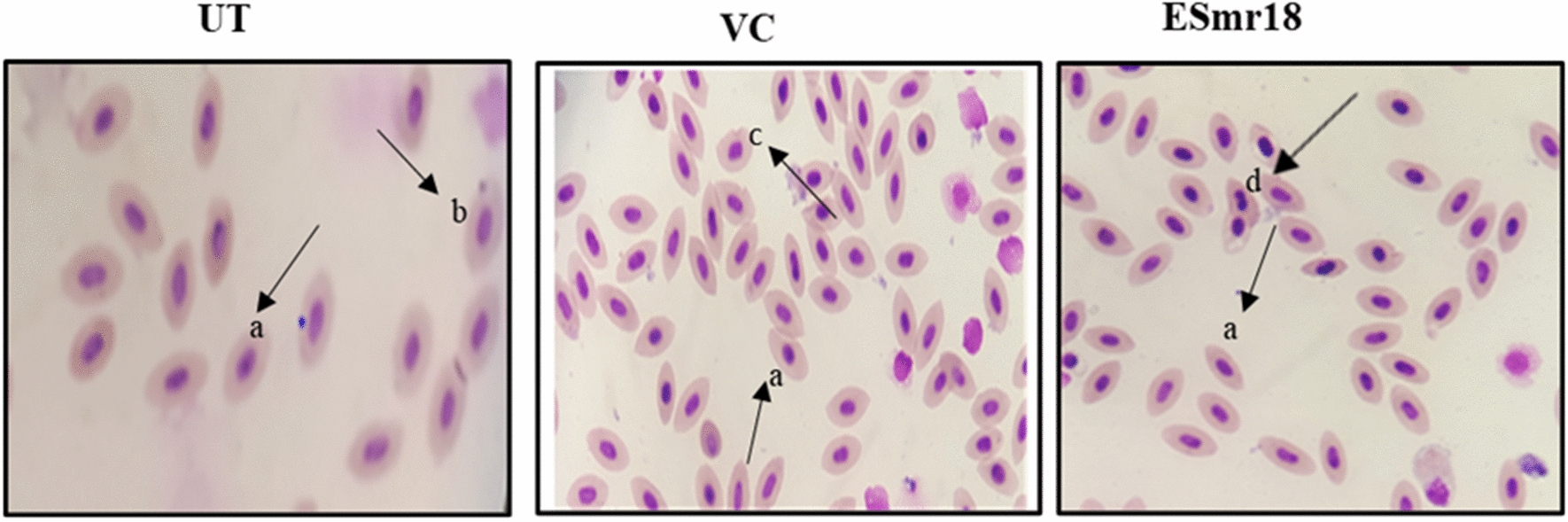
Nucleo-cellular abnormalities in the blood cells of *C. mrigala* (a) normal cells (b) micronuclei (c) necrotic cell (d) lobed nucleus

The micronucleus test is a nucleo-cellular abnormality assay. This test is used in toxicological studies for screening of genotoxic compounds for observing various types of aberrant cells (AC) like micronuclei, necrotic cells, lobed nucleus, and notched nucleus. Mean frequency of AC in the UT, VC and crude ESmr18 treated groups were 51 ± 7.549, 54 ± 7.937 and 59.6 ± 8.020/10,000 cells, respectively. Similarly, micronuclei frequency in UT, VC and crude ESmr18 treated group was 2.33 ± 0.577, 2.33 ± 0.577 and 2.66 ± 0.577. Non-significant differences were observed in AC and micronuclei cell frequency between the three groups (Fig. 8).

## Discussion

Animal-derived foods, such as poultry, and seafood, are susceptible to easy bacterial spoilage due to high water activity, favourable pH, and high nutrient content. Pathogenic microorganisms such as *E. coli*, *Salmonella* spp., *Campylobacter* spp., and *L. monocytogenes* can be commonly isolated from chicken and meat (Bohaychuk et al. 2006) that caused several food borne outbreaks (Morton et al. 2019; Mead et al. 2006; Nørrung and Buncic 2008). Thus, to prevent infections, raw animal-foods are treated with various chemical preservatives such as chlorine, nitrites, sodium chlorite and hypochlorite etc. These chemicals cause oxidative reactions in meat leading to adverse changes in the nutrient quality and the taste of the food. Secondly, they are also known to result in the formation of carcinogenic compounds in the treated food (Honikel 2008). Thus, safe bio preservatives are highly warranted.

Natural preservatives such as LAB and their bacteriocins (Yu et al. 2021) are being explored for food preservation and in antimicrobial food packaging systems. Number of studies have shown the applications of *Lactobacillus* spp. and their bacteriocins in food preservation for inhibiting the growth of Gram-positive pathogenic bacteria such as *Listeria* spp. (Woraprayote et al. 2018). However, the use of bacteriocinogenic strains and bacteriocins in food for inhibiting *Salmonella* spp. has not been explored much. This is probably because most of the bacteriocins secreted by LAB do not have activities against Gram-negative bacteria including *Salmonella* spp. Among LAB bacteriocins, few enterocins exhibit broad-spectrum activities against both Gram-positive and Gram-negative bacteria (Kasimin et al. 2022; Sharma et al. 2021) and therefore should be explored as food preservative. *Enterococci* spp. are commonly isolated from fermented cheese (Centeno et al. 1996) and used as starter cuture in other milk products (Wessels et al. 1990; Giraffa et al. 1997), where they impart characteristic flavour. Some strains of *E. faecium* such as SF68 etc. are also being used as probiotics for improving the health of humans and livestock (Franz et al. 2011).

In this study, *E. faecium* Smr18 and its enterocin ESmr18 was evaluated for its potential as an antimicrobial agent in food packaging and biopreservation of chicken, respectively. The nonpathogenicity of the isolate *E. faecium* Smr18, was determined by evaluating its susceptibility to antibiotics. Antibiotic susceptibility profile of the *Enterococcal* isolate can be used to differentiate the commensal nonpathogenic (belonging to clade B) isolates of enterococci from the clinical isolates that belong to clade A as 80% of the pathogenic clinical *E. faecium* strains are vancomycin-resistant and 90% are resistant to ampicillin (Hidron et al. 2008; Lebreton et al. 2013). *E. faecium* Smr18 was tested for its antibiotic susceptibility profile by using Kirby Bauer disk diffusion method that revealed that the strain was susceptible to the antibiotics ampicillin, penicillin-G, vancomycin, and erythromycin (data not shown). Further, the strain Smr18 secretes enterocin in the CS as evidenced by complete abrogation of its antimicrobial activity after treatment with proteolytic enzymes. On the other hand, catalase and lipase treatment had no effects on its antimicrobial activity. Further, we purified enterocin ESmr18 of 3.8 kDa that has antimicrobial activity against both Gram-positive and Gram-negative pathogens. Unlike other LAB bacteriocins that mostly inhibit Gram-positive bacteria, enterocins are known to inhibit Gram-negative bacteria also. Anti-*salmonella* activity of few enterocins from *E. faecium* has been reported previously. Enterocin B purified from *E. faecium* por1 had molecular weight of 7.2 kDa and it inhibited *S. typhi* and *S. enterica* (Ankaiah et al. 2018). Similarly, enterocin E-760 having molecular weight of 5.3 kDa inhibited several strains of *S. enterica* (Line et al. 2008)*.*

Further, the mode of action of the purified ESmr18 was studied that showed that the treatment of *S. enterica* and *L. monocytogenes* cells with purified ESmr18 at the MIC value (3.2 µg/ml) altered the cell membrane permeability in both the cases that resulted in efflux of potassium ions in the extracellular medium. The concentration of potassium ions peaked faster (10 min) in case of *L. monocytogenes* as compared to *S. enterica* cells, where the peak was obtained at 20 min. This can be explained by the presence of outer cell membrane in the cell wall of Gram-negative bacteria that acts as barrier to the enterocin. Similarly, bacteriocin produced by *L. plantarum* i.e., plantaricin MG caused peak efflux of potassium ions from *S. enterica* cells at 30 min (Gong et al. 2010). In another study, lacticin was shown to disrupt the cell membrane permeability of *L. monocytogenes* resulting in rapid efflux of potassium ions that peaked at 2.5 min (McAuliffe et al. 1998). However, in a recent study enterocin HDX-2 at 1X MIC (5 µg/ml) was shown to cause maximum efflux of potassium ions from *L. monocytogenes* cells at 160 min (Du et al. 2022).

In this study, *E. faecium* was immobilized in sodium alginate film and the film was tested for the first time against *S. enterica*-infected chicken. Sodium alginate is a polysaccharide produced by brown algae that is edible, non-toxic, biodegradable and bio compatible (Stephen et al. 2016). An added advantage of storing meat products in alginate films is that it prevents the surface drying along with weight loss in meat during storage (Silva et al. 2022). Our results showed that there was constant reduction in the CFU counts of *Salmonella* in *E. faecium* immobilized film as compared to control film with time. Maximum reduction of 3.0 log_10_ CFU was observed on 34th day of storage at 7–8 °C. Our results are in accordance with previously reported study, wherein, 3.0 log_10_ reduction in counts of *L. monocytogenes* was reported by sodium alginate film containing *Carnobacterium *spp. on day 28 (Concha-Meyer et al. 2011). Silva et al. (2022) also reported 1.2 log_10_ CFU reduction of *L. monocytogenes* on 8th day of storage in alginate film containing *Lactococcus lactis* and *Lc. garvieae* as compared to control.

Physico-chemical stability studies revealed that the antimicrobial activities of both CS and the crude enterocin was stable at pH ranging from 4 to 8 and at temperatures upto 100 °C. Crude enterocin could resist autoclaving for 40 min. Further, we studied the stability of crude ESmr18 in water and sodium acetate buffer at 7 °C. As the antimicrobial activity of crude ESmr18 dissolved in sodium acetate buffer was stable at 7–8 °C for 6 months, we tested its effect on *S. enterica* counts in chicken meat stored at 7 °C. Results showed a significant decrease ranging between 2.9 log_10_ CFU to 3.9 log_10_ CFU in *Salmonella* counts in enterocin-treated chicken samples as compared to untreated controls at all time points starting as soon as 1 h of the treatment till day 35. Similar to our studies, Ananou et al. (2010) showed significant reduction in the counts of *S. enterica* (2 log_10_ CFU) and *L. monocytogenes* (1.87 log_10_ CFU) on day 10 following addition of purified enterocin AS-48 in fermented sausage, fuet. However, in another study, AS-48 alone had no effect on the CFU counts of *S*. *enterica* inoculated as a cocktail of 5 different strains in Russian salad (Cobo Molinos et al. 2009). In another study, a formulation containing enterocins A and B used as preservative to sausages inoculated artificially with *S. enterica* at 3 log_10_ CFU and stored at 7 °C did not inhibit the growth of *S. enterica* (Jofre et al. 2009).

Next, the biosafety of ESmr18 was tested in an in vitro and an in vivo assay in a fish model. ESmr18 treatment of human RBCs resulted in 4.8% hemolysis at the highest tested dose of 4.98 µg/ ml. The lytic effect of ESmr18 was lower than that reported previously for other enterocins S37 (74.2% at 10 µg/ ml; Belguesmia et al. 2011) and P40 (19% at 2.5 µg/ ml; Vaucher et al. 2010a) but comparable to that caused by nisin (6% at 3.35 µg/ ml; Shin et al. 2015) and P34 (5.84% at 2.5 µg/ ml; Vaucher et al. 2010b).

Further, in vivo safety of orally administered ESmr18 was tested as it is a low molecular weight peptide, and it is known that small molecules of less than 4 kDa size can easily transit gut epithelial barrier (Dreyer et al. 2019). Fish is a popular model to study the in vivo toxicity of chemicals as it shows rapid response to chemicals, and the results of the experiment could be fairly extrapolated to humans (Demicco et al. 2010). *C. mrigala* was used for the in vivo experiments as it is readily available locally, and the biochemical profile of its sera is well studied (Ghayyur et al. 2021). Our results showed that oral administration of crude ESmr18 for four days to fish at a dose of 700 µg did not cause any significant changes in the liver and kidney biochemistry of the fish as compared to the vehicle-treated and normal control. Further, acute dosing of partially purified ESmr18 did not induce any genotoxicity in fish as shown by micronucleus and comet assay. Both these assays detect DNA damage in several tissues from one specimen at the same time. Similar to our studies, the toxicity of enterocin AS-48 was studied in zebra fish and Balb/c mouse model Cebrián et al. (2019). The study showed that the maximum tolerated dose at which no lethality was observed was 10 µg/ml. In mouse model, intraperitoneal injection of AS-48 at a high dose of 500 µg/g induced an alteration in biochemical parameters that reverted back to normal within 7 days. Baños et al. (2019) administrated 100 µg/ ml AS-48 to trout fish for 96 h and observed no toxicity or apparent signs of stress. The nontoxicity of ESmr18 combined with its broad spectrum activity makes it a promising candidate for use as safe biopreservative in foods stored under refrigeration conditions. Further experiments are required to determine the maximum tolerated doses of ESmr18 in animal models before its approval.

In conclusion, our study showed that *E. faecium* Smr18 secretes 3.8 kDa enterocin that inhibits pathogens by altering the cell membrane permeability. Enterocinogenic strain Smr18 was used in antimicrobial food packaging system that was shown for the first time to inhibit *Salmonella* contamination in chicken meat stored at refrigeration temperature (7–8 °C). The sodium alginate film used for the immobilisation of enterococci allowed the diffusion of the enterocin in the chicken samples that effectively inhibited the growth of *Salmonella* for 34 days. The direct addition of crude ESmr18 was equally efficient in inhibiting the growth of *S. enterica* in chicken samples stored at 7–8 °C for nearly 1 month. Further, ESmr18 did not cause hemolysis in human RBCs and was found safe when orally administered at high doses to fish.

## Supplementary Information

**Additional file 1: Figure S1.** Phylogenetic tree of *E. faecium* Smr18 and 21 other *Enterococcus* strains was constructed with *E. coli* as outgroup by using MEGA6 software. Using the Neighbour-Joining approach, the evolutionary history was deduced. The evolutionary history of the species studied is shown by the bootstrap consensus tree generated from 500 repetitions. The evolutionary distances were calculated by using the Maximum Composite Likelihood technique. **Figure S2.** SDS-PAGE showing resolved bands. Lane-1 protein marker, Lane-3 purified ESmr18. **Figure S3.** (A) Alginate film (B) Antimicrobial activity of alginate film with *E. faecium* and alginate film without *E. faecium* Smr18 cells against *S. enterica* as demonstrated by zone of inhibition on agar spot assay. **Figure S4.** Hemolytic activity of purified ESmr18 at different concentrations. The error bars show the standard deviation of three separate experiments conducted in triplicate. **Table S1.** physico-chemical characteristics of CS and ESmr18.

## Data Availability

Data will be made available on request.

## Abbreviations

CS: Culture supernatant
CFU: Colony-forming unit
RBC: Red blood cell
MRS: De Man Rogosa and Sharpe
BHI: Brain heart infusion
LAB: Lactic acid bacteria
NCBI-BLAST: National Center for Biotechnology Information: Basic Local Alignment Search Tool
NCIM: National Collection of Industrial Microorganisms
MTCC: Microbial Type Culture Collection
HEPES: 4-(2-Hydroxyethyl)-1-piperazineethanesulfonic acid
OD: Optical density
NaCl: Sodium chloride
AU: Arbitrary units
VC: Vehicle control
UC: Untreated control
TM: Tail moment
TL: Tail length
%TDNA: Percent tail DNA
SDS-PAGE: Sodium-dodecyl polyacrylamide gel electrophoresis

## References

[CR1] Agriopoulou S, Stamatelopoulou E, Sachadyn-Król M, Varzakas T (2020). Lactic acid bacteria as antibacterial agents to extend the shelf life of fresh and minimally processed fruits and vegetables: quality quality and safety aspects. Microorganisms.

[CR3] Ananou S, Garriga M, Jofré A, Aymerich T, Gálvez A, Maqueda M, Martínez-Bueno M, Valdivia E (2010). Combined effect of enterocin AS-48 and high hydrostatic pressure to control food-borne pathogens inoculated in low acid fermented sausages. Meat Sci.

[CR4] Ankaiah D, Palanichamy E, Antonyraj CB, Ayyanna R, Perumal V, Ahamed SI, Arul V (2018). Cloning, overexpression, purification of bacteriocin enterocin-B and structural analysis, interaction determination of enterocin-A, B against pathogenic bacteria and human cancer cells. Int J Biol Macromol.

[CR5] Baños A, Ariza JJ, Nuñez C, Gil-Martínez L, García-López JD, Martínez-Bueno M, Valdivia E (2019). Effects of *Enterococcus faecalis* UGRA10 and the enterocin AS-48 against the fish pathogen *Lactococcus garvieae*. Studies in vitro and in vivo. Food Microbiol.

[CR6] Belguesmia Y, Madi A, Sperandio D, Merieau A, Feuilloley M (2011). Growing insights into the safety of bacteriocins the case of enterocin S37. Res Microbiol.

[CR7] Bohaychuk VM, Gensler GE, King RK, Manninen KI, Sorensen O, Wu JT, Stiles ME, McMullen LM (2006). Occurrence of pathogens in raw and ready-to-eat meat and poultry products collected from the retail marketplace in Edmonton, Alberta, Canada. J Food Prot.

[CR8] Bradford MM (1976). A rapid and sensitive method for the quantitation of microgram quantities of protein utilizing the principle of protein-dye binding. Anal Biochem.

[CR66] Cebrián Rubén, Rodríguez-Cabezas M. Elena, Martín-Escolano Rubén, Rubiño Susana, Garrido-Barros María, Montalbán-López Manuel, Rosales María José, Sánchez-Moreno Manuel, Valdivia Eva, Martínez-Bueno Manuel, Marín Clotilde, Gálvez Julio, Maqueda Mercedes (2019). Preclinical studies of toxicity and safety of the AS-48 bacteriocin. Journal of Advanced Research.

[CR9] Callewaert R, Hugas M, De VL (2000). Competitiveness and bacteriocin production of Enterococci in the production of Spanish-style dry fermented sausages. Int J Food Microbiol.

[CR10] Centeno JA, Menéndez S, Rodríguez-Otero JL (1996). Main microbial flora present as natural starters in Cebreiro raw cow’s-milk cheese (Northwest Spain). Int J Food Microbiol.

[CR11] Chai SJ, Cole D, Nisler A, Mahon BE (2017). Poultry: the most common food in outbreaks with known pathogens, United States, 1998–2012. Epidemiol Infect.

[CR12] Cleveland J, Montville TJ, Nes IF, Chikindas ML (2001). Bacteriocins: safe, natural antimicrobials for food preservation. Int J Food Microbiol.

[CR13] Cobo Molinos A, Lucas López R, Abriouel H, Ben Omar N, Valdivia E, Gálvez A (2009). Inhibition of *Salmonella enterica* cells in deli-type salad by enterocin AS-48 in combination with other antimicrobials. Probiotics Antimicrob Proteins.

[CR14] Concha-Meyer A, Schöbitz R, Brito C, Fuentes R (2011). Lactic acid bacteria in an alginate film inhibit *Listeria monocytogenes* growth on smoked salmon. Food Control.

[CR15] Deegan LH, Cotter PD, Hill C, Ross P (2006). Bacteriocins: biological tools for bio-preservation and shelf-life extension. Int Dairy J.

[CR16] Demicco A, Cooper KR, Richardson JR, White LA (2010). Developmental neurotoxicity of pyrethroid insecticides in zebrafish embryos. Toxicol Sci.

[CR17] Dominguez SA, Schaffner DW (2009). Survival of *Salmonella* in processed chicken products during frozen storage. J Food Prot.

[CR18] Dreyer L, Smith C, Deane SM, Dicks LMT, van Staden AD (2019). Migration of bacteriocins across gastrointestinal epithelial and vascular endothelial cells, as determined using in vitro simulations. Sci Rep.

[CR60] Du Renpeng, Ping Wenxiang, Ge Jingping (2022). Purification, characterization and mechanism of action of enterocin HDX-2, a novel class IIa bacteriocin produced by Enterococcus faecium HDX-2. LWT.

[CR19] Franz CMAP, Huch M, Abriouel H, Holzapfel W, Gálvez A (2011). Enterococci as probiotics and their implications in food safety. Int J Food Microbiol.

[CR21] Geis A, Singh J, Teuber M (1983). Potential of lactic streptococci to produce bacteriocin. Appl Environ Microbiol.

[CR22] Ghayyur S, Khan MF, Tabassum S, Ahmad MS, Sajid M, Badshah K, Khan MA, Ghayyur S, Khan NA, Ahmad B, Qamer S (1822). A comparative study on the effects of selected pesticides on hemato-biochemistry and tissue histology of freshwater fish *Cirrhinus mrigala* (Hamilton, 1822). Saudi J Biol Sci.

[CR23] Giraffa G (2003). Functionality of enterococci in dairy products. Int J Food Microbiol.

[CR24] Giraffa G, Carminati D, Neviani E (1997). Enterococci isolated from dairy products: a review of risks and potential technological use. J Food Prot.

[CR61] Gong Han-Sheng, Meng Xiang-Chen, Wang Hui (2010). Mode of action of plantaricin MG, a bacteriocin active against Salmonella typhimurium. Journal of Basic Microbiology.

[CR26] Hidron AI, Edwards JR, Patel J, Horan TC, Sievert DM, Pollock DA, Fridkin SK (2008). Antimicrobial-resistant pathogens associated with healthcare-associated infections: annual summary of data reported to the national healthcare safety network at the centers for disease control and prevention, 2006–2007. Infect Control Hosp Epidemiol.

[CR27] Honikel KO (2008). The use and control of nitrate and nitrite for the processing of meat products. Meat Sci.

[CR28] Jofré A, Aymerich T, Garriga M (2009). Improvement of the food safety of low acid fermented sausages by enterocins A and B and high pressure. Food Control.

[CR29] Karkey A, Thwaites GE, Baker S (2018). The evolution of antimicrobial resistance in *Salmonella typhi*. Curr Opin Gastroenterol.

[CR62] Kasimin Melisa Elsie, Shamsuddin Suriyani, Molujin Arnold Marshall, Sabullah Mohd Khalizan, Gansau Jualang Azlan, Jawan Roslina (2022). Enterocin: Promising Biopreservative Produced by Enterococcus sp.. Microorganisms.

[CR31] Lebreton F, van Schaik W, Manson McGuire A, Godfrey P, Griggs A, Mazumdar V, Corander J, Cheng L, Saif S, Young S, Zeng Q (2013). Emergence of epidemic multidrug-resistant *Enterococcus faecium* from animal and commensal strains. Mbio.

[CR32] Line JE, Svetoch EA, Eruslanov BV, Perelygin VV, Mitsevich EV, Mitsevich IP, Levchuk VP, Svetoch OE, Seal BS, Siragusa GR, Stern NJ (2008). Isolation and purification of enterocin E-760 with broad antimicrobial activity against Gram-positive and Gram-negative bacteria. Antimicrob Agents Chemother.

[CR33] Massey RC, Lees D (1992). Surveillance of preservatives and their interactions in foodstuffs. Food Addit Contam.

[CR34] Maurício E, Rosado C, Duarte MP, Verissimo J, Bom S, Vasconcelos L (2017). Efficiency of nisin as preservative in cosmetics and topical products. Cosmetics.

[CR63] McAuliffe Olivia, Ryan Maire P., Ross R. Paul, Hill Colin, Breeuwer Pieter, Abee Tjakko (1998). Lacticin 3147, a Broad-Spectrum Bacteriocin Which Selectively Dissipates the Membrane Potential. Applied and Environmental Microbiology.

[CR35] McMullen LM, Stiles ME (1996). Potential for use of bacteriocin-producing lactic acid bacteria in the preservation of meats. J Food Prot.

[CR36] Mead PS, Dunne EF, Graves L, Wiedmann M, Patrick M, Hunter S, Salehi E, Mostashari F, Craig A, Mshar P, Bannerman T (2006). Nationwide outbreak of listeriosis due to contaminated meat. Epidemiol Infect.

[CR37] Medved ‘ova A, Koňuchová M, Kvočiková K, Hatalová I, Valík L (2020). Effect of lactic acid bacteria addition on the microbiological safety of pasta-flata types of cheeses. Front Microbiol.

[CR38] Mokoena MP, Omatola CA, Olaniran AO (2021). Applications of lactic acid bacteria and their bacteriocins against food spoilage microorganisms and foodborne pathogens. Molecules.

[CR39] Morton VK, Kearney A, Coleman S, Viswanathan M, Chau K, Orr A, Hexemer A (2019). Outbreaks of *Salmonella* illness associated with frozen raw breaded chicken products in Canada, 2015–2019. Epidemiol Infect.

[CR40] Nørrung B, Buncic S (2008). Microbial safety of meat in the European union. Meat Sci.

[CR41] Paiva AD, de Oliveira MD, de Paula SO, Baracat-Pereira MC, Breukink E, Mantovani HC (2012). Toxicity of bovicin HC5 against mammalian cell lines and the role of cholesterol in bacteriocin activity. Microbiol.

[CR42] Papagianni M, Anastasiadou S (2009). Pediocins: the bacteriocins of Pediococci. Sources, production, properties and applications. Microb Cell Fact.

[CR43] Piper JD, Piper PW (2017). Benzoate and sorbate salts: a systematic review of the potential hazards of these invaluable preservatives and the expanding spectrum of clinical uses for sodium benzoate. Compr Rev Food Sci.

[CR44] Ryan MP, O’Dwyer J, Adley CC (2017). Evaluation of the complex nomenclature of the clinically and veterinary significant pathogen *Salmonella*. Biomed Res Int.

[CR64] Sharma Preeti, Kaur Sumanpreet, Chadha Bhupinder Singh, Kaur Raminderjit, Kaur Manpreet, Kaur Sukhraj (2021). Anticancer and antimicrobial potential of enterocin 12a from Enterococcus faecium. BMC Microbiology.

[CR45] Shin JM, Ateia I, Paulus JR, Liu H, Fenno JC (2015). Antimicrobial nisin acts against saliva derived multi-species biofilms without cytotoxicity to human oral cells. Front Microbiol.

[CR46] Silva S, Ribeiro SC, Teixeira JA, Silva CCG (2022). Application of an alginate-based edible coating with bacteriocin-producing *Lactococcus* strains in fresh cheese preservation. Lwt.

[CR47] Sindelar JJ, Milkowski AL (2011) Sodium nitrite in processed meat and poultry meats: a review of curing and examining the risk/benefit of its use. American Meat Sci Association White Paper Series vol 3, pp 1–4

[CR48] Smadi H, Sargeant JM, Shannon HS, Raina P (2012). Growth and inactivation of *Salmonella* at low refrigerated storage temperatures and thermal inactivation on raw chicken meat and laboratory media: mixed effect meta-analysis. J Epidemiol Glob Health.

[CR49] Soltani S, Hammami R, Cotter PD, Rebuffat S, Said LB, Gaudreau H, Bédard F, Biron E, Drider D, Fliss I (2021). Bacteriocins as a new generation of antimicrobials: toxicity aspects and regulations. FEMS Microbiol Rev.

[CR50] Stephen AM, Phillips GO, Williams PA (2016). Food polysaccharides and their applications.

[CR51] Trujillo IZ, Quiroz C, Gutierrez MA, Arias J, Renteria M (1991). Fluoroquinolones in the treatment of typhoid fever and the carrier state. Eur J Clin Microbiol Infect Dis.

[CR52] Vaucher AR, De Souza, A, Brandelli A (2010). Evaluation of the in vitro cytotoxicity of the antimicrobial peptide P34. Cell Biol Int.

[CR53] Vaucher RA, Teixeira M, Brandelli A (2010). Investigation of the cytotoxicity of antimicrobial peptide p40 on eukaryotic cells. Curr Microbiol.

[CR65] Wessels Delille, Jooste P.J., Mostert J.F. (1990). Technologically important characteristics of Enterococcus isolates from milk and dairy products. International Journal of Food Microbiology.

[CR54] Woraprayote W, Pumpuang L, Tosukhowong A, Zendo T, Sonomoto K, Benjakul S, Visessanguan W (2018). Antimicrobial biodegradable food packaging impregnated with Bacteriocin 7293 for control of pathogenic bacteria in pangasius fish fillets. LWT.

[CR55] World Health Organization. Typhoid (2018) Typhoid https://www.who.int/health-topics/typhoid#tab=tab_1. Accessed 24 April 2022.

[CR56] York A (2022). Emergent *Enterococcus* toxins. Nat Rev Microbiol.

[CR57] Yu HH, Chin YW, Paik HD (2021). Application of natural preservatives for meat and meat products against food-borne pathogens and spoilage bacteria: a review. Foods.

[CR58] Yun SH, Lee SW, Koo HN, Kim GH (2014). Assessment of electron beam-induced abnormal development and DNA damage in *Spodoptera litura* (F.) (Lepidoptera: Noctuidae). Radiat Phys Chem.

[CR59] Zhou L, van Heel AJ, Montalban-Lopez M, Kuipers OP (2016). Potentiating the activity of nisin against *Escherichia coli*. Front Cell Dev Biol.

