# Supplementary material for: Biopreservative efficacy of Enterococcus faecium-immobilised film and its enterocin against Salmonella enterica
Source: AMB Express. 2023 Jan 23;13:11. doi: 10.1186/s13568-023-01516-z (PMC9871141; doi:10.1186/s13568-023-01516-z)
Supplement: Supplementary file 1 — Additional file 1: Figure S1. Phylogenetic tree of E. faecium Smr18 and 21 other Enterococcus strains was constructed with E. coli as outgroup by using MEGA6 software. Using the Neighbour-Joining approach, the evolutionary history was deduced. The evolutionary history of the species studied is shown by the bootstrap consensus tree generated from 500 repetitions. The evolutionary distances were calculated by using the Maximum Composite Likelihood technique. Figure S2. SDS-PAGE showing resolved bands. Lane-1 protein marker, Lane-3 purified ESmr18. Figure S3. (A) Alginate film (B) Antimicrobial activity of alginate film with E. faecium and alginate film without E. faecium Smr18 cells against S. enterica as demonstrated by zone of inhibition on agar spot assay. Figure S4. Hemolytic activity of purified ESmr18 at different concentrations. The error bars show the standard deviation of three separate experiments conducted in triplicate. Table S1. physico-chemical characteristics of CS and ESmr18. [file 13568_2023_1516_MOESM1_ESM.docx]

**Figure S1.** Phylogenetic tree of *E. faecium* Smr18 and 21 other *Enterococcus* strains was constructed with *E. coli* as outgroup by using MEGA6 software. Using the Neighbour-Joining approach, the evolutionary history was deduced. The evolutionary history of the species studied is shown by the bootstrap consensus tree generated from 500 repetitions. The evolutionary distances were calculated by using the Maximum Composite Likelihood technique.


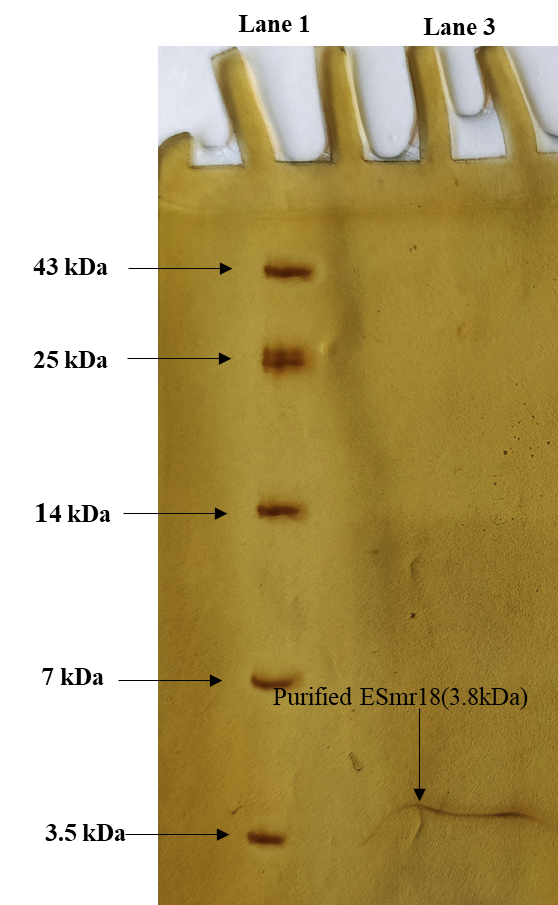


**Figure S2.** SDS-PAGE showing resolved bands. Lane-1 protein marker, Lane-3 purified ESmr18

**Figure S3. (A)** Alginate film **(B)** Antimicrobial activity of alginate film with *E.faecium* and alginate film without *E. faecium* Smr18 cells against *S. enterica* as demonstrated by zone of inhibition on agar spot assay.

**Figure S4.** Hemolytic activity of purified ESmr18 at different concentrations. The error bars show the standard deviation of three separate experiments conducted in triplicate.

Table S1 physico-chemical characteristics of CS and ESmr18

|  | Ph | | | | | Temp ˚C | | | | |
| --- | --- | --- | --- | --- | --- | --- | --- | --- | --- | --- |
|  | 2 | 4 | 6 | 8 | 10 |  | 60 | 80 | 100 | Autoclaving |
| CS | - | 14 | 15 | 12 | - |  | 16 | 15 | 10 | - |
| ESmr | - | 16 | 19 | 15 | - |  | 18 | 13 | 11 | 10 |
